# Skin temperature change in patients with meibomian gland dysfunction following intense pulsed light treatment

**DOI:** 10.3389/fmed.2022.893940

**Published:** 2022-08-10

**Authors:** Jeongseop Yun, Ji Sang Min

**Affiliations:** Department of Ophthalmology, Kim's Eye Hospital, Konyang University College of Medicine, Seoul, South Korea

**Keywords:** dry eye, intense pulsed light therapy, skin temperature, meibomian gland dysfunction, vessel ablation

## Abstract

**Purpose:**

We investigated the change in skin temperature of treated areas during intense pulsed light (IPL) treatment in patients who have meibomian gland dysfunction (MGD) to determine whether there is superficial telangiectatic blood vessel ablation.

**Methods:**

The medical records of 90 patients (90 eyes) with MGD who underwent IPL treatment were reviewed. The patients had undergone IPL treatment four times every 4 weeks. Ocular Surface Disease Index (OSDI) scores, dry eye (DE), and MGD parameters were obtained before the first and after the fourth IPL treatments. The skin temperatures of the upper and lower lids were measured before every IPL treatment.

**Results:**

The skin temperatures of the lower lids were 31.89 ± 0.72°C at the first IPL (IPL#1), 30.89 ± 0.63°C at the second IPL (IPL#2), 30.14 ± 0.95°C at the third IPL (IPL#3), and 29.74 ± 0.87°C at the fourth IPL (IPL#4) treatments. The skin temperatures of upper lids were 32.01 ± 0.69°C at IPL#1, 31.13 ± 0.75°C at IPL#2, 30.34 ± 1.07°C at IPL#3, and 29.91 ± 0.76°C at IPL#4. The skin temperature of the upper and lower lids significantly decreased with every IPL treatment. Schirmer 1 test (ST) result was 12.97 ± 10.22 mm before IPL#1 and 14.45 ± 9.99 mm after IPL#4. Tear break-up time (TBUT) was 3.15 ± 1.38 s before IPL#1 and 5.53 ± 2.34 s after IPL#4. Corneal staining scores (CFS) was 1.61 ± 3.09 before IPL#1 and 0.50 ± 0.78 after IPL#4. Lipid layer thickness (LLT) was 71.88 ± 26.34 nm before IPL#1 and 68.38 ± 24.16 nm after IPL#4. Lid margin abnormality score (LAS) was 1.96 ± 0.62 before IPL#1 and 0.86 ± 0.67 after IPL#4. Meibum expressibility (ME) was 1.67 ± 0.87 before IPL#1 and 1.03 ± 1.67 after IPL#4. Meibum quality (MQ) was 18.18 ± 6.34 before IPL#1 and 10.16 ± 5.48 after IPL#4. OSDI was 35.38 ± 19.97 before IPL#1 and 15.48 ± 34.32 after IPL#4. OSDI scores, DE, and MGD parameters significantly improved after the fourth IPL treatment but not ST and LLT.

**Conclusion:**

Our study showed that the occurrence of superficial telangiectatic vessels were indirectly reduced by the decrease in skin temperature accompanying IPL treatments in patients with MGD.

## Introduction

Meibomian gland dysfunction (MGD) is a disease occurring in the meibomian glands, characterized by ductal obstruction or changes in the quantity and quality of meibomian gland secretions (1). As a consequence, changes in meibomian gland secretions can lead to an unstable tear film (2). Symptoms such as dryness, eye irritation, foreign body sensations, burning sensations, tearing, and fatigue may be experienced by such changes (3). Warm compresses, lid massages, use of antibiotic and anti-inflammatory ointments, and artificial tears are known conventional treatments for MGD (4). However, despite the variety of treatment options currently available, many patients with MGD do not respond to treatments. In other words, symptoms may not disappear completely or for the long-term. This has led to the rise of intense pulsed light treatment (IPL) (5).

IPL therapy has been applied for the removal of hirsutism, pigmented lesions, and vascular lesions like cavernous hemangiomas, venous malformations, telangiectasia, and port wine stains (6). The first application of IPL therapy in the field of ophthalmology was performed by Toyos et al. (7), who found that patients with facial rosacea had significant improvements in dry eye (DE) symptoms after IPL treatment. Studies have shown that IPL therapy is effective in improving both subjective symptoms and objective findings in patients with mild to moderate MGD or DE (8–11).

The mechanisms underlying IPL treatment in MGD patients have been postulated to involve superficial blood vessel destruction, meibum fluidification, epithelial turnover downregulation, photomodulation, and antimicrobial effects (12). Despite these proposed mechanisms, the action mechanism of IPL in MGD and DE patients remains obscure, and there is no common consensus about the actual action mechanism (12). Gan et al. (13) used IPL therapy to treat patients with facial telangiectasia and reported that superficial blood vessels were ablated and that the patients' skin temperatures decreased after IPL treatment. In addition, a reduction in facial telangiectasia after IPL treatment has been shown to decrease local blood flow, thereby lowering skin temperature.

The purpose of this study was to investigate whether superficial vascular resection occurs during IPL treatment in MGD patients, by measuring their skin temperatures at the treatment site.

## Materials and methods

### Patients

This study was performed with the approval of the relevant institutional review board (No. KEH 2021-11-015-002). We reviewed the medical records of patients diagnosed with MGD from March 2021 to December 2021 who had received four IPL treatments. Patients with MGD were diagnosed according to previous criteria (14, 15): (i) there had to be at least one symptom from a list including ocular fatigue, discharge, foreign body sensation, dryness, uncomfortable sensation, sticky sensation, pain, epiphora, itching, redness, heavy sensation, glare, excessive blinking, burning sensation, and ocular discomfort upon arising; (ii) at least one abnormal lid margin finding associated with vascular engorgement, anterior or posterior replacement of the mucocutaneous junction, and irregularity; and (iii) plugged meibomian gland orifices and poor meibum expressibility (ME) in the target eye. IPL treatment was performed in patients who were refractory to conventional treatments such as artificial tears, warm compresses, eyelid scrubs, or topical/systemic antibiotics. Inclusion criteria of the enrolled patients were as follows: (i) age of more than 18 years and (ii) completion of four sessions of IPL treatment at 4-week intervals. Patients meeting the following criteria were excluded: patients who had (i) missing DE and MGD evaluations before the first IPL treatment or after the fourth IPL treatment; (ii) systemic disorders that may have affected DE or MGD disease; (iii) undergone oral or topical retinoid use; (iv) intraocular surgery in the past 6 months; (v) receipt of botulinum toxin or filler injections in the past month; (vi) uncontrolled ocular disease; or (vii) dark skin type, such as Fitzpatrick skin type V or VI (16); (viii) previous diagnosis of rosacea.

### IPL procedure

**Figure 1:**
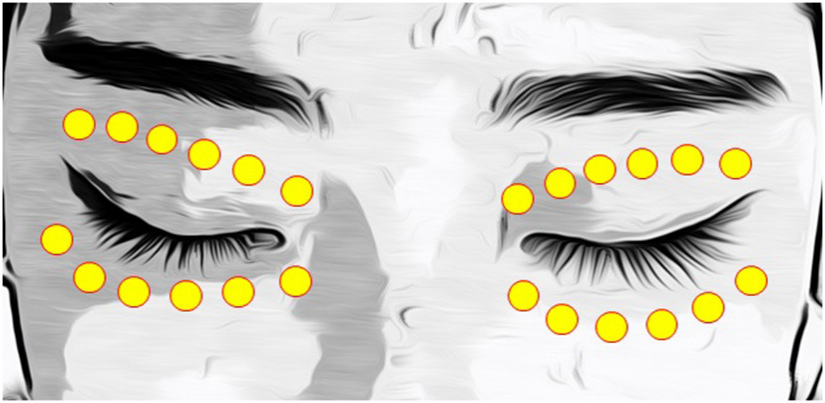
IPL treatment zone, including six periocular areas on each eyelid. IPL, intense pulsed light.

Prior to IPL treatment, patients were advised to clean their face to remove any makeup. After the ultrasound gel was applied to the eyelid skin area, the clinician placed the Jaeger lead plate (Katena Products, Denville, NJ, USA) within the conjunctival sac to protect the eye. The M22 Optima device (Lumenis, Yokneam, Israel) was used, and a duration of 6.0 ms and an interval of 60.0 ms were set. Furthermore, a 590-nm filter and a 6-mm cylindrical light guide were used on the hand piece (17). The fluence was set up according to the Fitzpatrick skin type (13–19 J/cm2), as reported in previous studies (17–19). Six IPL pulses were applied to each the upper and lower eyelids (Figure 1) (17, 18). At the end of IPL treatment, meibomian gland expression was performed using an Arita Meibomian Gland Compressor (Katena Products, Denville, NJ, USA).

### Skin temperature measurement before IPL treatment

**Figure 2:**
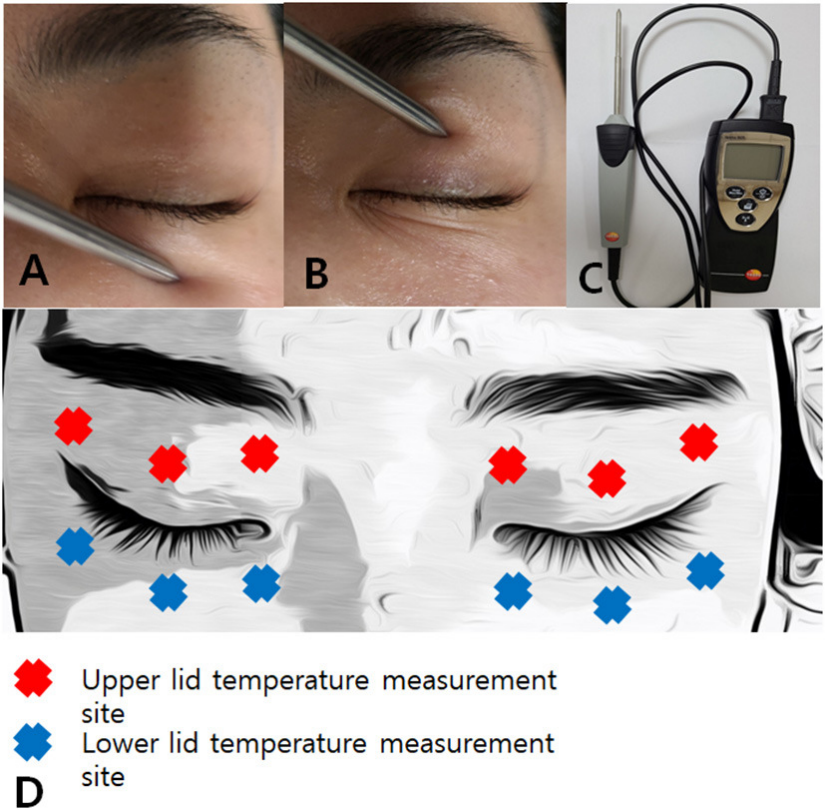
A picture of skin temperature measurement of lower lid **(A)** and upper lid **(B)** and a picture of a thermometer (Testo 925, one-channel temperature measuring instrument T/C Type K, Testo AG, Germany) **(C)**. Skin temperature measurement site including three periocular areas of the upper and lower lids **(D)**.

Patients rested for 20 min at 22–24°C and 50–60% humidity prior to each IPL session (13). The skin temperatures of the upper (Figure 2A) and lower (Figure 2B) eyelids were measured using a thermometer (Testo 925, one-channel temperature measuring instrument T/C Type K, Testo AG, Germany)(Figure 2C) before each IPL session at the first, second, third, and fourth IPL treatments (IPL#1 to IPL#4) (13). The temperatures of each eyelid were measured in the temporal, middle, and nasal areas (Figure 2D), and the average temperature of each eyelid was calculated from the three measurements.

### Clinical assessment

**Figure 3:**
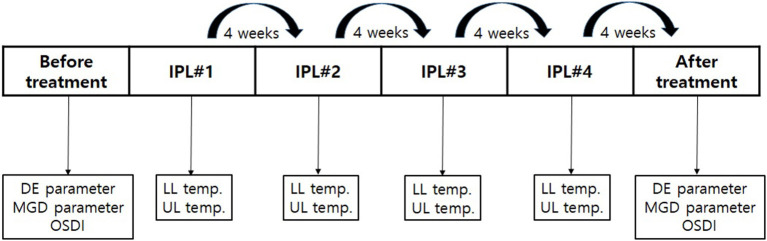
The schedule of IPL treatment and clinical measurements. DE and MGD parameters and the OSDI were measured before the first IPL treatment and after the fourth IPL treatment. The skin temperatures were measured before every IPL treatment. IPL, intense pulsed light; DE, dry eye; MGD, meibomian gland dysfunction; OSDI, Ocular Surface Disease Index. IPL, intense pulsed light; DE, dry eye; IPL#1, first intense pulsed light treatment; IPL#2, second intense pulsed light treatment; IPL#3, third intense pulsed light treatment; IPL#4, fourth intense pulsed light treatment; MGD, meibomian gland dysfunction; OSDI, Ocular Surface Disease Index; LL, lower eyelid; UL, upper eyelid.

DE and MGD parameters, as well as Ocular Surface Disease Index (OSDI) scores were measured before IPL#1 and after IPL#4 in all patients (Figure 3).

DE parameters such as the Schirmer 1 test (ST), tear break-up time (TBUT) test, corneal staining scores (CFSs), and MGD parameters such as meibomian gland examinations, their lipid layer thickness (LLT), and lid margin abnormality score (LAS) were obtained. Standard paper strips (Eagle Vision, Memphis, TN, USA) were placed on one-third of the mid-lateral portions of the lower fornix without topical anesthesia for ST (17). After 5 min, the length of the wet columns were recorded in millimeters (17). A single fluorescein strip (Haag-Streit International, Koniz, Switzerland) wetted with a drop of preservative-free normal saline was placed over the inferior tear meniscus, and then the CFSs and TBUT were measured (17). Three repeated measurements of the TBUT, after several blinks, were obtained, and the average was calculated (17). The corneal staining was acquired according to the criteria of the Oxford Schema (20). LLT was obtained by using the LipiView interferometer (TearScience, Morrisville, NC, USA) (17). The lid margins and meibomian glands were examined under slit lamp microscopy after all the other measurements had been obtained (17). LAS was assigned as 0 (absent) or 1 (present) for lid margin irregularity, vessel engorgement, plugged meibomian glands, and anterior or posterior mucocutaneous junction displacement (21). The meibum expression level (ME) was determined by applying digital pressure to the five glands of the lower eyelid, and classifying them as follows: grade 0, all five glands expressible; grade 1, three to four glands expressible; grade 2, one to two glands expressible; and grade 3, none of the glands expressible (21). Meibum quality (MQ) was also examined and designated one of the following scores: grade 0, clear; grade 1, cloudy; grade 2, cloudy with granular debris; and grade 3, toothpaste-like. A total score was obtained by summing the scores for the eight glands (maximum score: 24) (21).

DE and MGD parameters of the right eye, as well as OSDI scores before IPL#1 and after IPL#4 were compared using paired *t*-tests. Additionally, the skin temperatures of the upper and lower eyelids at IPL#1, IPL#2, IPL#3, and IPL#4 were compared using repeated-measure analyses of variance.

The amount of the change of the upper and lower eyelids temperature at IPL#1 and at IPL#4 was obtained, and the amount of the changes of each DE and MGD parameters between before IPL#1 and after IPL#4 were obtained. Additionally, multivariate linear regression analysis was performed to reveal the relation between skin temperature change and improvements of DE and MGD parameters.

## Results

### Patient demographics

A total of 90 patients were included in this study (90 eyes, 24 men, and 66 women). The average age of the patients was 54.67 ± 13.62 years.

### Skin temperature of each IPL session

Table 1 shows the skin temperature changes and average skin temperature of the lower and upper eyelids of the patients at IPL#1, IPL#2, IPL#3, and IPL#4. The skin temperatures of lower eyelids were 31.89 ± 0.72°C at IPL#1, 30.89 ± 0.63°C at IPL#2, 30.14 ± 0.95°C at IPL#3, and 29.74 ± 0.87°C at IPL#4. The skin temperatures of upper eyelids were 32.01 ± 0.69°C at IPL#1, 31.13 ± 0.75°C at IPL#2, 30.34 ± 1.07°C at IPL#3, and 29.91 ± 0.76°C at IPL#4. The average temperatures of the upper and lower eyelids were 31.95 ± 0.70°C at IPL#1, 31.01 ± 0.70°C at IPL#2, 30.24 ± 1.01°C at IPL#3, and 29.83 ± 0.82°C at IPL#4. The temperatures of the upper and lower eyelids, including the average temperature, were significantly lower after all sessions than before the first IPL session.

**Table 1:** Changes in the skin temperature (°C) of eyelids after each intense pulsed light treatment.

	**IPL#1**	**IPL#2**	**IPL#3**	**IPL#4**	**Difference** **IPL#1– IPL#2**	**Difference** **IPL#1–IPL#3**	**Difference** **IPL#1–IPL#4**	**Difference** **IPL#2–IPL#3**	**Difference** **IPL#3–IPL#4**
LL	31.89 ± 0.72	30.89 ± 0.63	30.14 ± 0.95	29.74 ± 0.87	<0.001	<0.001	<0.001	<0.001	0.001
UL	32.01 ± 0.69	31.13 ± 0.75	30.34 ± 1.07	29.91 ± 0.76	<0.001	<0.001	<0.001	<0.001	<0.001
Average of LL and UL	31.95 ± 0.70	31.01 ± 0.70	30.24 ± 1.01	29.83 ± 0.82	<0.001	<0.001	<0.001	<0.001	<0.001

IPL, intense pulsed light; IPL#1, first intense pulsed light treatment; IPL#2, second intense pulsed light treatment; IPL#3, third intense pulsed light treatment; IPL#4, fourth intense pulsed light treatment; LL, Lower Eyelid; UL, Upper Eyelid.

### Comparison of DE and MGD parameters and OSDI scores before IPL#1 and after IPL#4

Table 2 shows the changes in the DE and MGD parameters, and in the OSDI scores of the patients before IPL#1 and after IPL#4. ST was 12.97 ± 10.22 mm before IPL#1 and 14.45 ± 9.99 mm after IPL#4. TBUT was 3.15 ± 1.38 s before IPL#1 and 5.53 ± 2.34 s after IPL#4. CFS was 1.61 ± 3.09 before IPL#1 and 0.50 ± 0.78 after IPL#4. LLT was 71.88 ± 26.34 nm before IPL#1 and 68.38 ± 24.16 nm after IPL#4. LAS was 1.96 ± 0.62 before IPL#1 and 0.86 ± 0.67 after IPL#4. ME was 1.67 ± 0.87 before IPL#1 and 1.03 ± 1.67 after IPL#4. MQ was 18.18 ± 6.34 before IPL#1 and 10.16 ± 5.48 after IPL#4. OSDI was 35.38 ± 19.97 before IPL#1 and 15.48 ± 34.32 after IPL#4. The TBUT, CFS, LAS, ME, and MQ measurements obtained before IPL#1 were significantly higher than those measured after IPL#4. There were no significant differences between the ST and LLT scores before IPL#1 and after IPL#4. The OSDI scores before IPL#1 decreased significantly when compared with those after IPL#4.

**Table 2:** Summary of the findings obtained before and after intense pulsed light treatments.

	**Before IPL#1**	**After IPL#4**	***P*-value**
ST	12.97, 10.22	14.45, 9.99	0.211
TBUT	3.15, 1.38	5.53, 2.34	<0.001
CFS	1.61, 3.09	0.50, 0.78	0.001
LLT	71.88, 26.34	68.38, 24.16	0.209
LAS	1.96, 0.62	0.86, 0.67	<0.001
ME	1.67, 0.87	1.03, 1.67	<0.001
MQ	18.18, 6.34	10.16, 5.48	<0.001
OSDI	35.38, 19.97	15.48, 34.32	<0.001

IPL#1, first intense pulsed light treatment; IPL#4, fourth intense pulsed light treatment; ST, Schirmer 1 Test; TBUT, Tear Break-Up Time; CFS, Corneal and Conjunctival Staining Scores; LLT, Lipid Layer Thickness; LAS, lid margin abnormality score; ME, Meibum Expressibility; MQ, Meibum Quality, OSDI, Ocular Surface Disease Index.

### Multivariate linear regression analysis between skin temperature change and DE and MGD parameter changes

There was no significant difference between LLT and ST before and after 4 sessions of IPL treatments, therefore multivariate linear regression analysis was not performed. Table 3 shows the result of multivariate linear regression analysis result between amount of skin temperature decrease and DE and MGD parameters except ST and LLT. Multivariate linear regression analysis showed that there was significant relation between the amount of skin temperature decrease of upper, lower, and average of upper and lower lid after 4 sessions of IPL treatments and CFS improvement. However, there were no significant relationship between the skin temp decrease and DE and MGD parameters except CFS.

**Table 3:** Multivariate linear regression analysis between skin temperature (°C) change and DE and MGD parameter improvement.

**LL temperature change**			**UL temperature change**			**Average of LL and UL temperature change**		
	**B**	***p*-value**		**B**	***p*-value**		**B**	***p*-value**
LAS	0.099	0.287	LAS	0.009	0.939	LAS	0.084	0.476
ME	−0.090	0.250	ME	−0.0743	0.483	ME	−0.107	0.284
MQ	−0.132	0.871	MQ	0.567	0.596	MQ	0.158	0.878
CFS	0.912	0.009	CFS	0.408	0.040	CFS	0.928	0.040
TBUT	−0.028	0.919	TBUT	−0.042	0.908	TBUT	−0.043	0.904
OSDI	−0.577	0.696	OSDI	0.071	0.975	OSDI	−0.514	0.816

LL, lower eyelid; UL, upper eyelid; TBUT, Tear Break-Up Time; LAS, Lid Margin Abnormality Score; ME, Meibum Expressibility; MQ, Meibum Quality; CFS, Corneal and Conjunctival Staining Scores; OSDI, Ocular Surface Disease Index.

## Discussion

We investigated whether there were changes in skin temperature associated with each IPL session. We found that patients with MGD who were treated with IPL experienced improvements in ocular discomfort as well as in their DE and MGD parameters. In addition, there was a gradual decrease and downward trend in skin temperature after each IPL session.

IPL was first introduced for the treatment of vascular diseases of the skin in 1976. The concept of photothermolysis was introduced in 1983, and a flash lamp for treating vascular lesions of the skin was developed in 1990 (12). In 1994, the first commercialized IPL machine was released by Lumenis (12), and was applied for the removal of hirsutism, pigmented lesions, and vascular lesions like cavernous hemangiomas, venous malformations, telangiectasia, and port wine stains in dermatology fields (6). In 2002, Toyos et al. discovered that dry eyes improved after IPL treatment in facial rosacea patients and introduced IPL into the ophthalmology field. Many studies have been conducted on the use of IPL treatments in patients with MGD (7, 8, 19, 21–25). These studies have shown that ocular discomfort, DE, and MGD parameters improved after IPL treatment (7, 8, 19, 21–25). Similar to previous studies, the current study also found that DE and MGD parameters, as well as OSDI scores, improved after IPL treatment. In addition, IPL treatment is effective in reducing eyelid ecchymosis after eye lid surgery (26), treatment for blepharokeratoconjunctivitis (27), and ocular demodex infestation (28).

Several studies have tried to prove the occurrence of superficial ablation in the field of dermatology (13, 29, 30). Bäumler et al. (29) presented a mathematical model for calculating the photon distribution and thermal effects of IPL emissions within cutaneous blood vessels. They demonstrated the occurrence of superficial vessel ablation resulting from IPL treatment. Furthermore, studies have reported that IPL treatment was effective in patients with MGD; superficial blood vessel destruction, meibum fluidification, epithelial turnover downregulation, photomodulation, antimicrobial effects, modulation of the secretion of pro- and anti-inflammatory molecules, and suppression of matrix metalloproteinases (MMPs) were proposed as possible mechanisms of IPL treatments of MGD patients in previous studies (12, 31). However, no study has demonstrated the mechanism of action of IPL treatment clearly. Therefore, there is a need for research to directly or indirectly prove the mechanism of action of IPL treatment in patients with MGD. To the best knowledge, the current study is the first attempt to prove vessel ablation on the treatment area in MGD patients indirectly.

Recently, Mejía et al. (31) demonstrated the concept that the main mechanism of action of IPL on the eyelids is secondary to its effects on the mitochondria of the tarsal plate. The light absorbed into the mitochondria in the tarsal plate activates the mitochondria, and exerts its initial effect, resulting in increased ATP production, modulation of reactive oxygen species, and induction of transcription factors. Together, these effects produce proliferation and increased cell migration in the acini of the meibomian glands, in addition to the modulation of cytokines, growth factors, and the levels of inflammatory mediators, and finally an increase in cell oxygenation. Therefore, research on the relationship between mitochondria activation mechanism and reduction in skin temperature at the IPL treatment site is considered necessary in the future.

Gan et al. (13) reported that IPL treatment was effective in patients with facial telangiectasia, and they confirmed a decrease in superficial vessel ablation and a reduction in skin temperature at the affected site after IPL treatment. In addition, it has been reported that the reduction of facial telangiectasia after IPL treatment reduced the local blood flow, and thus the skin temperature (13). Additionally, Su et al. (32) reported that local inflammation in MGD patients may increase local blood flow on the eyelid and result in increases in the eyelid skin temperature. In the current study, a gradual decrease in skin temperature was observed after successive IPL treatments, thus indirectly confirming the occurrence of superficial vessel ablation. Further studies are required to confirm the occurrence of superficial vessel ablation after IPL treatment in patients with MGD by applying mathematical models (29) or by evaluating the presence of direct superficial ablation.

Several studies have reported the relationship between skin temperature and MGD. Most of them have demonstrated that the use of eyelid warming devices was effective in patients with MGD (33–35). These studies showed that the skin temperatures of patients with MGD were approximately 33.2°C (33) and 32.7°C (35), which are higher than the eyelid temperature at IPL#1 in the current study. In previous studies, skin temperature was measured using an infrared thermometer (33, 35). However, in the current study, the skin temperature was measured using a contact thermometer. The differences in the skin temperatures between the current study and the previous studies may be attributed to the use of different measuring devices. Gan et al. (13) measured skin temperature with the same thermometer as was used in the current study and obtained temperature values that are almost identical to those obtained in the current study. Many studies have measured the skin temperature of patients with MGD, but the current study is the first to investigate the changes in skin temperature after IPL treatment in patients with MGD.

One study measured eyelid temperature using an infrared thermometer and found that the eyelid temperature of patients with MGD was higher than the temperature of the controls (32). In addition, this study concluded that the accumulation of inflammatory molecules on the ocular surface of patients with MGD might increase their skin temperature. In the current study, the skin temperature of patients with MGD gradually decreased, and the signs and symptoms of MGD improved after serial IPL treatments. In addition, there was significant relation between the skin temperature decrease and CFS. Therefore, there is a possibility that the degree of change in skin temperature due to IPL treatment is related to the degree of improvement in MGD. Changes in skin temperature following IPL treatment could also be a predictor of the response to MGD treatment. Additional studies should be conducted to further explore the changes in skin temperature due to IPL therapy and the degree of MGD treatment. Furthermore, studies are required to investigate the relationship between changes in skin temperature following IPL treatment and changes in inflammatory substances on the ocular surface.

In this study, it was confirmed that the eyelid temperature of patients gradually decreased after IPL treatment. However, it was not clear whether the eyelid temperature change was a result of vessel ablation or a result of decreased inflammation of the eyelids and ocular surface. However, Su et al. (32) confirmed that the eyelid skin temperature was high in MGD patients, and Gan et al. (13) reported that the skin temperature at the treatment site dropped after IPL treatment. All these previous studies support the notion that vessel ablation at the IPL treatment site in MGD patients resulted in decreased skin temperature at the treatment area in this current study. However, this study was a retrospective study and could not directly confirm vessel ablation or decrease of inflammation on the eyelid or ocular surface. Therefore, additional studies are needed in the future to directly ascertain the relationship between eyelid skin temperature change and vessel ablation or eyelid and/or ocular surface inflammation in MGD patients in response to IPL treatment.

This study has certain limitations which should be considered. First, it is retrospective. Second, it was difficult to directly confirm the occurrence of superficial vessel ablation on the eyelids of patients with MGD in an ophthalmology clinic. Additional studies that directly confirm the occurrence of superficial vessel ablation on eyelids or that apply mathematical models are required. Third, the follow-up period was limited to 4 weeks after the final treatment. Longer follow-up periods are needed to evaluate long-term changes in a patient's eyelid skin temperature. Furthermore, randomized controlled clinical trials or well-designed cohort studies are required to confirm the occurrence of superficial vessel ablation on the eyelids of patients with MGD after IPL treatment.

In conclusion, the reduction of superficial telangiectatic vessels was confirmed indirectly through a decrease in skin temperature after IPL treatments in patients with MGD. Therefore, further evaluations of the relationship between skin temperature changes and MGD improvement are required.

## Data availability statement

The raw data supporting the conclusions of this article will be made available by the authors, without undue reservation.

## Ethics statement

The studies involving human participants were reviewed and approved by Kim Eye Hospital Institutional Review Board. Written informed consent for participation was not required for this study in accordance with the national legislation and the institutional requirements.

## Author contributions

Conceptualization, design, and critical revisions were performed by JY and JM. Data acquisition and drafting of the manuscript were performed by JY. Data/statistical analyses and interpretation and supervision were performed by JM. Both authors approved the final version of the manuscript.

## Conflict of interest

The authors declare that the research was conducted in the absence of any commercial or financial relationships that could be construed as a potential conflict of interest.

## Publisher's note

All claims expressed in this article are solely those of the authors and do not necessarily represent those of their affiliated organizations, or those of the publisher, the editors and the reviewers. Any product that may be evaluated in this article, or claim that may be made by its manufacturer, is not guaranteed or endorsed by the publisher.
